# Students’ views of mentoring at Bahria University Medical and Dental College

**DOI:** 10.12669/pjms.326.10625

**Published:** 2016

**Authors:** Ambreen Usmani, Quratulain Omaeer

**Affiliations:** 1 Prof. Dr. Ambreen Usmani, Professor & HOD-Anatomy, Dyp Director-Medical Education, Bahria University Medical and Dental College, Karachi, Pakistan; 2 Dr. Quratulain Omaeer, Senior Lecturer of Anatomy, Bahria University Medical and Dental College, Karachi, Pakistan

**Keywords:** Mentoring, Mentor, Mentee, Medical students, Multicultural

## Abstract

**Objective:**

To explore the mentoring program on a subset of Pakistani medical students in a private medical college.

**Method:**

Total students targeted were 300 MBBS students of 1^st^ Year (group B), 2^nd^ Year (group C) and 3^rd^ Year (group D), of these 256 students filled the self-reported questionnaire. The questionnaire was based on Likert Scale. The statements in the questionnaire are designed in a positive manner so that if the students agree to them the level of satisfaction with the mentoring program was considered significantly good. Open-ended questionnaires were also given so as to have a clearer concept of the students’ perception. This study is a mixed method study catering to both quantitative and qualitative domains.

**Results:**

The overall results reported that the junior students of group B and group C showed higher satisfaction in being mentored as compared group D (p-value=0.001). All three groups were compared with each other to check the response of every statement by applying Tukey’s test. Analysis of the result showed that majority of the students considered mentoring program a beneficial tool for their academic and non-academic lives. The students of all three years have reported that the mentor is mostly available and helps to reconcile internal conflicts. They also confirmed that their mentor keeps records but most of the students have reported that communication via email is limited.

**Conclusion:**

Majority of students of Bahria University Medical and Dental College are able to carry on with their academic and non-academic routine due to the presence of mentoring. The medical students appreciated the presence of a mentor during thick and thin; they have also accepted that it is due to the presence of this guide that they are able to continue with their difficult studies in these difficult times.

## INTRODUCTION

It was in the era of Greek mythology when the concept of mentor came into being. Penelope, wife of Greek god Odysseus gave birth to a baby boy, Telemachus who grew up without any guidance and support from his father due to his non availability. At that time Odysseus entrusted his son’s upbringing to Athena, the goddess of wisdom. According to Greek mythology it was Athena’s constant mentoring that transformed Telemachus from a lame, shy boy into a young successful man.^1^,^2^

Mentoring is communicating in a non-threatening environment to guide and facilitate a mentee by his/her mentor. A mentor is someone of higher rank, ideally older in age than the mentee for this relationship to be successful.^3^,^4^ Mentoring of medical students started in USA from 1990 onwards^4^ and is in progress till today. It is now gaining a lot of attention in Pakistan as there is more awareness of this strategy.

In Pakistan we have diverse cultures being a country with many provinces. These provinces have their own distinct cultures and languages.^5^ Within these languages there are some variation in the speaking style of the individuals depending upon the city or village of their residence. The students in our country undergo 12 or 13 years of schooling and then enter into a medical college. When our students enter a professional college the average age is 18±1.27 year, students from all over the provinces apply and attain admission on the basis of merit.^6^

At Bahria University Medical and Dental College a diverse group of students from all provinces enter the college every year. Being from such different cultures and backgrounds they have a lot of adjustment problems, in order to combat their academic and non-academic situations, a structured mentoring program began, which has been in place since the inception of this college.^7^,^8^ The mentors have to look into the psychological domain of each mentee also, for which our mentors are trained via continuous workshops and are assigned 10 students who stay with them till they pass out of college after 5 years.^9^

In spite of several hurdles the medical students slowly and steadily improve themselves due to the guidance provided by the mentoring program, the role of the mentors is to identify their mentees’ strengths and weaknesses. Those mentees who have problems which are very sensitive try to shy away from their issues. This may become problematic and literature research shows that the strenuous studies of medicine may lead a student to commit suicide at the maximum.^10^ The mentor identify students who are facing difficulties by several mentoring sessions. Once these individuals are identified they are counseled and names are given to the supervisor of the mentoring program.^11^

Along with these hurdles other associated problems are a part of our daily routine, the mentees gets so use to sharing everything with his/her mentor. Simply venting out helps them get through another day. Mentoring sessions are helpful for the students as well as the mentors as mentioned in several studies.^11^,^12^

Mentors have heavy responsibilities on their shoulders and are trained to empathize and listen but at times it becomes very difficult for the mentor to keep emotions away from their duty. As the suicide rate of medical students is raising it is even more essential that such programs are introduced and the mentor play their roles effectively and honestly. The mentors cannot solve some of the non-academic issues of the students but their presence and listening skills help their mentees to grow from an unconfident individuals to smart and confident doctors ready to enter the world with sound level of knowledge, skills and attitude.^12^ To explore the mentoring program on a subset of Pakistani medical students in a public medical college.

## METHODS

Total students targeted were 300 MBBS students of 1^st^ Year (group B), 2^nd^ Year (group C) and 3^rd^ Year (group D), of these 256 students filled the self-reported questionnaire which was anonymous and collected by the support staff. The questionnaire was based on Likert Scale with strongly agree (SA) as 5, agree (A) as 4, neutral (N) as 3, disagree (DA) as 2 and strongly disagree (SDA) as 1. The statements in the questionnaire was designed in a positive manner so that if the students agree to them the level of satisfaction with the program was considered significantly good. Open-ended questionnaires were also given so as to have a clearer conception of the students’ perception which were divided into themes and the answers grouped under each theme. This study is a mixed method study catering to both quantitative and qualitative domains

### Statistical analysis

The data was entered and analyzed by SPSS version 15. The total variables were assessed by applying Chi-square and each individual statement was analyzed by applying Tukey’s test. P-value ≤ 0.05 is considered significant.

## RESULTS

**Fig.1:**
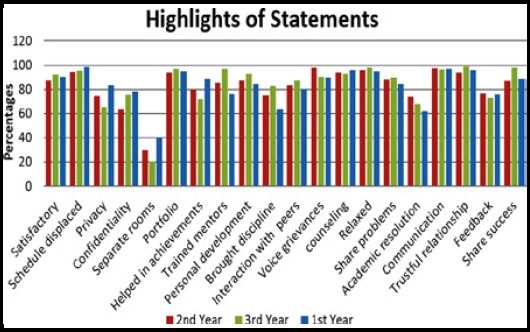
Students perception graph.

**Fig.2:**
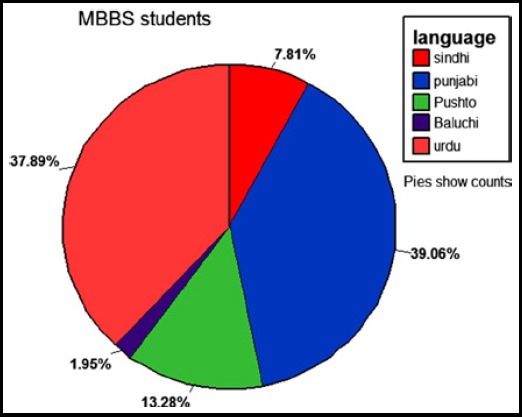
Ethnicity and mother tongue of students showing multicultural atmosphere.

The overall results reported that the junior students of group B and group C showed higher satisfaction and well-being after being mentored as compared group D. Chi-square is applied showing a significant p-value of 0.001 (Table-I). All three groups were compared with each other to check the response of every statement by applying Tukey’s test. However students of all three years have reported that the mentor is mostly available and helps to reconcile internal conflicts. They also confirmed that their mentor keeps records but most of the students have reported that communication via email is limited (Fig.1). The frequencies of different languages are shown in Fig.2.

**Table-I:** Mentoring leading to satisfaction and well-being.

Group	n	SA n (%)	A n (%)	N n (%)	DA n (%)	SDA n (%)	Total responses	p-value
B	85	167 (39.29)	140(32.94)	80(18.82)	28 (6.5)	10 (2.3)	425	0.001
C	109	344(63.11)	153(28.07)	22(4.03)	20(3.66)	6 (1.1)	545	
D	62	33 (10.64)	130 (41.93)	120 (38.7)	14 (46.66)	13(4.19)	310	

Total		544 (42.5)	423(33.04)	222(17.34)	62(4.84)	29(2.26)	1280

The open-ended questions consisted of five themes which were responsibility, personality, confidentiality, privacy and trust. The questions were designed in accordance with these theme. The open ended questions were grouped under these themes and accumulatively the answers received were:

***Q1. Why is mentoring important for you?***

“It helps me vent out”

“I know that someone is always there to help me”

“Mentoring has made me a better student and a confident individual”

***Q2. How has mentoring developed your personality?***

“I have improved in dressing”

“I have been able to overcome my shyness”

“There has been no change in my personality”

***Q3. How do you react when your mentor is unable to solve your problem?***

“I feel angry”

“I can understand that all my problems cannot be solved everyone has limitations”

***Q4. Do you have issues of confidentiality and privacy?***

“We are taken into different rooms for mentoring sessions”

“I can openly express my thoughts”

***Q5. Do you confide all your academic and non-academic issues to your mentor?***

“I usually discuss academic issues”

“I trust my mentor but she should have more power to solve our non-academic issues”

“I have no particular issue but I am satisfied talking to my mentor”

## DISCUSSION

Mentoring is required by students of all fields of life. It’s not an easy task for the young individuals to start their career in medicine without the help of a mentor^13^ that is why mentoring plays a key role for a flourishing career in academic medicine.^14^ While mentorship is provided at an individual level, the advantages are observed to be fruitful both for the individual and the organization.^15^ Mentoring is still not part of all medical colleges in Pakistan. At Bahria University Medical and Dental College (BUMDC) mentoring was implemented since the establishment of this college. Usmani et al. conducted a research in 2010 at this college regarding the perception and effect of mentoring on mentors in the mentorship program.^16^ It was found out that mentorship program foster affirmative change in mentees and the mentors were highly satisfied and contented performing this noble task.

Mentoring programs should be evaluated from time to time by the faculty of mentors as well as mentees.^17^ Mentoring at BUMDC follows the inclusion criteria as mentioned by Meinal^18^ which includes that all students should be a part of the mentoring program. The authors mention that for the mentor to establish a relationship with the mentee, this program should be for a minimum of one year with no maximum limit. The program is made not only to make the mentees excel academically, but also to support the overall development of mentees. The study has been divided into themes around which the questionnaire was developed

Mentors create an environment in which mentees realize their potentiality as a successful professional person.^19^ It’s important for good mentors to retain the goal of this relationship and keep focus on mentee’s personal and professional growth over time.^20^ Borch et al. research indicated that students are dependent on their mentors for provision of ideas of professional development and they expect their mentors to act as councilors for them in attaining their goals.^20^ Most of our mentees are of the opinion that mentoring has facilitated professional development and helped them improve their academic progress. Mentees described their mentors as the individuals providing both emotional and psychological support. This quality of mentor was stated as the most valued feature of mentor mentee relationship.^21^ In our study it was revealed that students have come from almost all cities/villages of Pakistan. Most of the students at the start of medical career were very shy and there was almost no social interaction, mentors set meetings with the mentee individually and in groups to make students interact with each other and help them to become gregarious. Mentors provide encouragement and support required by the students for improving learning habits, interpersonal dealings, communication skills, problem solving strategies and positive attitude.^22^

Straus et al. mentioned that effectual mentoring relationships require dedication and meeting with mentees at regular intervals.^23^ Good quality mentoring relationships need active maintenance. It was found out through our research that most of the mentees are of the opinion that their mentors give them sufficient time whenever they are in need of it.

Confidentiality is an essential part of mentoring relationship, keeping mentee’s information private is the platform for a trusting relationship where your mentee feels secure enough to talk openly without hesitation.^23^ Majority of the students at BUMDC felt that their mentors are trust worthy. Although mentorship is usually provided at an individual level, the benefits are seen both at the individual and organizational levels.

A mentor is a person who can provide guidance and answer questions; he/she is there to provide suggestions to the mentee.^24^ It was clearly evident from our research that mentors have dedicatedly achieved this task. They are excellent councilors and great source of professional advice for the young individuals.

Mentoring is a platform where mentees can come and voice their grievances and mentor is there to listen to their problems and encourage reflection so that mentee are empowered in making their own decisions.^24^ Our research shows that mentee here are getting the opportunity to discuss their problems and mentors act as an excellent listener. Rehman et al. mentioned in their research that the students’ identified their college mentors an important element to maintain their wellbeing.^22^

Relationship assistance by mentors for solving educational and personal issues signifies that the structure of the mentoring program supported the development of an effective mentor/mentee relationship.^14^ Mentors play an imperative role in developing ethics, professionalism, values and the talent of medicine that cannot be learned from text. It has been observed that mentors also provide emotional support when mentees feel down. This affiliation benefits mentors also, through greater efficiency, personal and career satisfaction. All of this brings in mentor mentee relationship awareness, mutual respect, and explicit communication about the relationship.^18^,^23^

Medical students have to cope with lots of work load and in order for them to have a balance in life they need to be regimented. To make students disciplined mentor certainly play an important role. Usmani A has reported that mentors are successful in attaining this goal.^24^

## CONCLUSION

In a multicultural setting, the medical student undergoing five years of strenuous studies have appreciated the rigorous mentoring program of the medical college. Majority of the students are able to carry on with their academic and non-academic routine in a stress free manner due to the presence of mentoring. The medical students appreciated the presence of a mentor during thick and thin.
